# Une déformation du fémur en flacon d'Erlenmeyer: la maladie de Gaucher

**DOI:** 10.11604/pamj.2015.20.418.5824

**Published:** 2015-04-29

**Authors:** Faten Frikha, Zouhir Bahloul

**Affiliations:** 1 Service de Médecine Interne, CHU Hédi Chaker, Sfax, Tunisie

**Keywords:** Maladie de Gaucher, bêta-glucocérébrosidase lysosomale, atteinte osseuse, Gaucher disease, lysosomal beta-glucocerebrosidase, atteinte osseuse

## Image en medicine

La maladie de Gaucher de type 1 (MG1) est une maladie multisystémique rare due à un déficit génétique en bêta-glucocérébrosidase lysosomale. Ses signes cliniques sont très polymorphes: splénomégalie (95%), hépatomégalie (80%), cytopénies (thrombopénie, anémie, neutropénie)… L'atteinte ostéoarticulaire est une complication majeure qui se voit dans 70 à 100% des cas selon les séries. Les anomalies du remodelage osseux induisent les classiques déformations distales des fémurs ou proximales des tibias en “flacon d'Erlenmeyer”, généralement de façon bilatérale et symétrique, avec une perte de la concavité de la région métaphysaire et un élargissement anormal de l'os à cet endroit. Un Homme âgé de 20 ans qui présente une MG1 diagnostiquée à l’âge de 4 ans était hospitalisé pour des douleurs osseuses diffuses. L'examen clinique objectivait une splénomégalie énorme allant jusqu'au pelvis, une hépatomégalie modérée et une douleur exquise à la palpation des épineuses lombaires. A la biologie, il existait une thrombopénie à 97000/mm^3^. La radiographie standard des fémurs montrait une déformation des tiers inférieurs des fémurs donnant l'aspect d'Erlenmeyer. L'IRM des confirmait l'infiltration médullaire des deux fémurs et du bassin avec des anomalies de signal en rapport avec des infarctus osseux.

**Figure 1:**
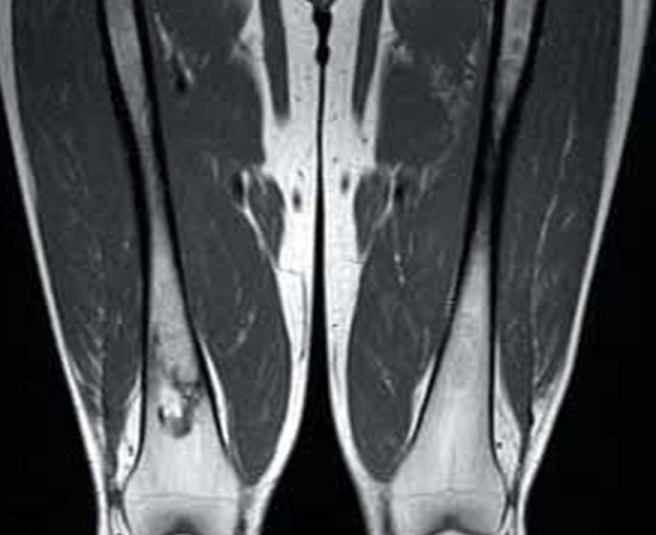
Coupe coronale des fémurs en pondération T2: déformation des tiers inférieurs des fémurs donnant l'aspect de flacon d'Erlenmeyer

